# The limitations of using simple definitions of glucocorticoid exposure to predict fracture risk: A cohort study

**DOI:** 10.1016/j.bone.2018.09.004

**Published:** 2018-12

**Authors:** Danielle E. Robinson, Tjeerd P. van Staa, Elaine M. Dennison, Cyrus Cooper, William G. Dixon

**Affiliations:** a Arthritis Research UK Centre for Epidemiology, School of Biological Sciences, Faculty of Biology, Medicine and Health, The University of Manchester, Oxford Road, Manchester M13 9PT, UK; b Health eResearch Centre, Farr Institute for Health Informatics Research, University of Manchester, Vaughan House, Portsmouth Road, M13 9PL, UK; c Utrecht University, Faculty of Science, Division of Pharmacoepidemiology and Clinical Pharmacology, Utrecht, the Netherlands; d MRC Lifecourse Epidemiology Unit, University of Southampton, Southampton General Hospital, Tremona Road, Southampton SO16 6YD, UK; e Victoria University, Wellington, New Zealand; f NIHR Musculoskeletal Biomedical Research Unit, Nuffield Department of Orthopaedics, Rheumatology and Musculoskeletal Sciences, University of Oxford, Oxford OX3 5UG, UK; g NIHR Nutrition Biomedical Research Centre, University of Southampton and University Hospital Southampton NHS Foundation Trust, Southampton General Hospital, Southampton SO16 6YD, UK; h NIHR Manchester Biomedical Research Centre, Manchester University, NHS Foundation Trust, Manchester Academic Health Science Centre, UK

**Keywords:** Glucocorticoids, Fracture, Epidemiology, Exposure definitions, Rheumatoid arthritis

## Abstract

**Purpose:**

To evaluate the effects of different definitions of glucocorticoid (GC) exposure on the magnitude and pattern of fracture risk using the same dataset.

**Methods:**

Data from patients with rheumatoid arthritis (RA) were extracted from the Clinical Practice Research Datalink, a primary care database with electronic health records in the United Kingdom. Patients exposed to oral GCs were matched to up to two unexposed patients by age, gender and location. The first osteoporotic fracture was identified and adjusted and unadjusted cox proportional hazard ratios (HR) and 95% confidence intervals (CI) produced for fracture risk following GC therapy using different models of risk attribution. These include models demonstrating the effect of dose, duration and recency of GC exposure.

**Results:**

There were 16,507 patients included. Exposed patients were older and had more comorbidities. GC therapy was associated with an increased risk of fracture, with the effect size influenced by risk attribution model. The risk of fracture decreased with less recent exposure from HR (95% CI) 1.66 (1.27, 2.16) during the first month of stopping GCs to 1.11 (0.79, 1.57) for between 1 and 3 months. The risk of fracture increased with current daily dose, HR 1.44 (1.17, 1.77) for 5–9.9 mg prednisolone equivalent dose (PEQ) to 3.02 (1.77, 5.15) for 15–19.9 mg PEQ. Risk of fracture increased with cumulative dose, a function of dose and duration, from HR 1.22 (1.03, 1.44) for <1 g to 1.83 (1.35, 2.48) for 7.5–10 g.

**Conclusion:**

GC exposure was associated with excess fracture risk, with effect size differing according to definition of exposure. This highlights the need to incorporate all exposure dimensions (dose, duration and recency) in these patient's fracture risk assessments.

## Highlights

• We compared definitions of glucocorticoid exposure and their associated risk of fracture.
• Dose, duration and recency of oral glucocorticoid exposure individually affect the risk of fracture.
• Dose, duration and recency of glucocorticoid exposure is not yet incorporated into fracture risk calculators.
• Clinicians should consider the combined effects of dose, duration and recency of exposure when assessing fracture risk.

## 1 Introduction

Glucocorticoids (GCs) have been used for over 65 years as an anti-inflammatory treatment for patients with rheumatoid arthritis (RA) [1]. They rapidly reduce inflammation and improve associated symptoms such as pain [2]. Oral GCs are used at least once by around half of RA patients, with 13% using them continuously for more than a year [3]. However, GCs have been associated with a number of adverse effects include diabetes, serious infection and fracture [4].

Fractures are a common adverse effect of oral GCs and carry substantial health burden and economic cost [5]. There is an increased risk of fractures in patients with RA with rates of 188 per 10,000 person years [6] compared to 78 per 10,000 person years in the general population [7]. In oral GC users, fracture rates are higher even when osteoporosis is not present [8,9]. This increase in the risk of fracture may be in part due to the effect of GCs on bone architecture, regeneration and remodelling [10].

To complicate the understanding of the effects on fracture risk, oral GCs are often prescribed in dynamic patterns where patients may be regularly changing dose and use GCs for different periods of time. These changing exposure patterns make it difficult to accurately analyse how risk is attributed from GC exposure to fracture risk using statistical models since different aspects of the exposure may affect the risk of fracture.

A recent literature review [11] of 38 papers of fracture risk in oral GC users with RA found that there were five commonly used methods of defining risk attribution of GCs to fracture risk, but that none of these methods simultaneously considered the exposure dimensions of dose, duration and recency of GC exposure. There was a marked heterogeneity in the fracture risk estimates, with results ranging from a protective effect to more than a three-fold increased risk of fracture. This broad range of risk estimates may be explained in part by these different exposure definitions, but may also be influenced by the population under study, exposure patterns, the comparator population, included confounders and study design. Unfortunately, none of these 38 studies assessed the impact of different exposure definitions on fracture risk using same dataset and study population.

Fracture risk assessments, such as FRAX, are now often used in daily clinical practice for estimating the risk of fracture and to determine the initiation of bone protection treatment. Most of the risk scores only consider GC use as a yes/no question, [[12], [13], [14], [15]] whilst some consider current daily dose [16]. None of these risk scores consider the patterns of GC use through time, therefore potentially estimating the fracture risk in oral GC users without considering a wide variability in risk with different doses and durations.

The primary aim of this work was to evaluate, in a single setting, the impact of using definitions of oral GC exposure on the prediction of fracture risk and to evaluate the associations between risk of fractures and the exposure characteristics of dose, duration and recency of exposure.

## 2 Methods

### 2.1 The clinical practice research Datalink

This study was conducted using data of the Clinical Practice Research Datalink (CPRD), a research database which contains anonymised electronic health records from 650 general practices in the United Kingdom (UK). It includes records on over 10 million patients, with about 8% of the UK population currently included in CPRD [17]. All clinical encounters within primary care are included in the CPRD with the primary reason for the consultation recorded and encoded using Read codes. Furthermore, any important clinical information sent from hospital to the general practitioner (GP) is usually recorded using medical codes. Additional relevant information within CPRD includes basic demographic information about the patient and practice, coded diagnoses, any prescribed medicines and hospital referrals. The CPRD has been found to be broadly representative of the UK in terms of age and sex [18]. Data are considered suitable for use in analyses when they are “up to standard”, where “up to standard” defines practices with data that meet the minimum quality criteria based on continuity in data recording and no longer collecting data about patients no longer at the practice. [19] At present there are over 10 million ‘up to standard’ records of current and previous patients.

### 2.2 Study design

Retrospective cohort study.

### 2.3 Study participants

Patients registered with CPRD between 1/1/1992 and 31/12/2014 were eligible for inclusion if they were over the age of 18, specified as male or female, and had a diagnosis of RA. A diagnosis of RA was identified using a validated algorithm [20]. The earliest date of eligibility was the latest of age 18, one year after the patient was registered in the practice, one year after the practice became ‘up to standard’ or the date of RA diagnosis. Patients were followed until the first of death, leaving the practice, the date of the last data collection for that practice or 31/12/2014.

The follow-up of each RA patient in the study population was classified according to GC exposure. GC prescriptions for all eligible patients were identified from the primary care prescription records. End dates of each prescription were estimated, using the quantity, prescribed number of tablets per day and number of days prescribed. The method for dealing with any missing information about prescription duration is explained in supplement 1. If patients had ever taken >100 mg prednisolone equivalent per day at any time, the patient was excluded from the analysis since such high dosages are unlikely in the treatment of RA.

Person time was divided into periods of non-exposure to GC (i.e., time prior to start of GC exposure or complete follow-up if never exposed to GCs), current and past exposure (see below for the definitions). Patients could move to different exposure categories over time. Risk set sampling was conducted to match each patient starting GC exposure (exposed) with up to two patients who were non-exposed to GCs at the time of the matching. Matching was done by age, gender and geographic location, with matching by geographical location done in a hierarchical manner. Patients were first matched on practice, then region of practice. If there was no match within the region, geographic location was discarded and the patient was only matched on age and gender. Patients who were exposed to GCs in the 6 months prior to a diagnosis of RA were also eligible for inclusion as an exposed patient. Matched non-exposed patients entered on their eligibility date whilst matched exposed patients entered on their first GC exposure. All patients had to have one year free from GCs prior to entry date, otherwise they were excluded from the analysis. Patients in the unexposed group contributed follow-up time until end of follow-up or start of GC exposure, whichever date came first. Exposure to GC therapy given by alternative routes of administration (e.g. topical, inhaled) prior to baseline was included as a covariate, with a category for each route of administration.

The outcome of interest was incident osteoporotic fracture. Fractures at any of the following six anatomic locations created the composite outcome of an osteoporotic fracture: hip, vertebra, forearm, humerus, ribs and pelvis. [21] The reporting of both vertebral and hip fractures have previously been validated in the CPRD. [22] No other location has yet been validated. Patients with a history of fracture at an unspecified location prior to baseline were excluded prior to entry to the exposed or unexposed cohort as we would be unable to ascertain whether a new fracture code represented an incident fracture. Patients with a history of fracture at one of the osteoporotic locations remained in the study but were followed until their first fracture at a different osteoporotic location with fractures at the same location assumed to be a repeat. I.e. if a patient had a history of hip fracture, only fractures at either the vertebra, forearm, humerus, ribs or pelvis would be considered an incident fracture. Unspecified fractures during follow-up did not affect the analysis since it was impossible to determine the location of the fracture, and hence whether to include or exclude it.

The following baseline information was extracted for each patient: year of birth, gender, body mass index, alcohol category, number of general practice contacts six months prior to cohort entry (a surrogate measure for disease severity), Charlson Co-morbidity Score, [23] history of any prior fracture, history of user of other administrations of GCs, baseline disease modifying anti-rheumatic drugs (DMARDs), drugs affecting bone mineral density (including calcium and vitamin D use, breast and prostate cancer treatments, and drugs for breast cancer and prostate cancer), and drugs affecting risk of falls (including opioids, anticonvulsants, antipsychotics and benzodiazepines). Use of anti-osteoporotic therapies (including bisphosphonates, denosumab, raloxifene and strontium ranelate) was included as a time varying covariate. If information about body mass index (BMI) or alcohol use was missing, an indicator of missingness was included.

### 2.4 Statistical methods

The exposed and unexposed categories were compared using Chi squared tests for categorical and binary variables, Mann-Whitney *U* tests for continuous variables which did not follow a normal distribution (GP contacts and follow up) and *t*-tests for continuous variables which followed a normal distribution (age at baseline).

Primary analyses assessed the impact of GCs on fracture risk in patients with RA using a range of different definitions for GC exposure in regression models. Model 1 represented the recency of exposure using the categories current use, use in the past 1 day to 1 month, use in the past 1 to 3 months, use in the past 3 to 6 months, use in the past 6 to 12 months, use >12 months ago and never use. Model 2 represented current daily dose with categories of never use, 0.1 to 4.9 mg prednisolone equivalent dose (PEQ)/day, 5 to 9.9 mg PEQ/day, 10 to 14.9 mg PEQ/day, 15 to 19.9 mg PEQ/day, 20 to 24.9 mg PEQ/day and >25 mg PEQ/day reported. Model 3 represented cumulative dose with categories for never use, 0.1 to 0.9 g PEQ, 1 to 2.4 g PEQ, 2.5 to 4.9 g PEQ, 5 to 7.4 g PEQ, 7.5 to 10 g PEQ and >10 g PEQ. Model 4 considered peak dose with categories for never use, 0.1 to 9.9 mg PEQ, 10 to 19.9 mg PEQ, 20 to 39.9 mg PEQ, 40 to 59.9 mg PEQ and 60 to 100 mg PEQ. These four models were tested individually since they reflect the range of models used in different analyses and thus allow us to consider the impact of model choice as well as exploring the effect of the aspects of discontinuation, current and cumulative dose (and hence duration of use) on the risk of fracture.

Exposure time was calculated as the person time spent in each category for each of the four models. Rates of fracture for each category within each model were calculated by dividing the number of fractures which occurred in that category by the total person years of exposure per category. Rates are reported per 10,000 person years of exposure.

Time-varying Cox proportional hazards models were used to estimate the effects of different exposure characteristics. Crude and multiple adjusted hazard ratios (HRs) were calculated in addition to 95% confidence intervals (CI). Adjustments were made by adding individual covariates initially, with age, gender and osteoporotic therapy use, to ensure the results were changing as expected, before all covariates were added. A priori confounders with a *p*-value < 0.2 were included in the final model. Data were analysed using Stata version 13.1.

This study was approved by the Independent Scientific Advisory Committee of the CPRD (protocol number 15_145R).

## 3 Results

**Fig. 1:**
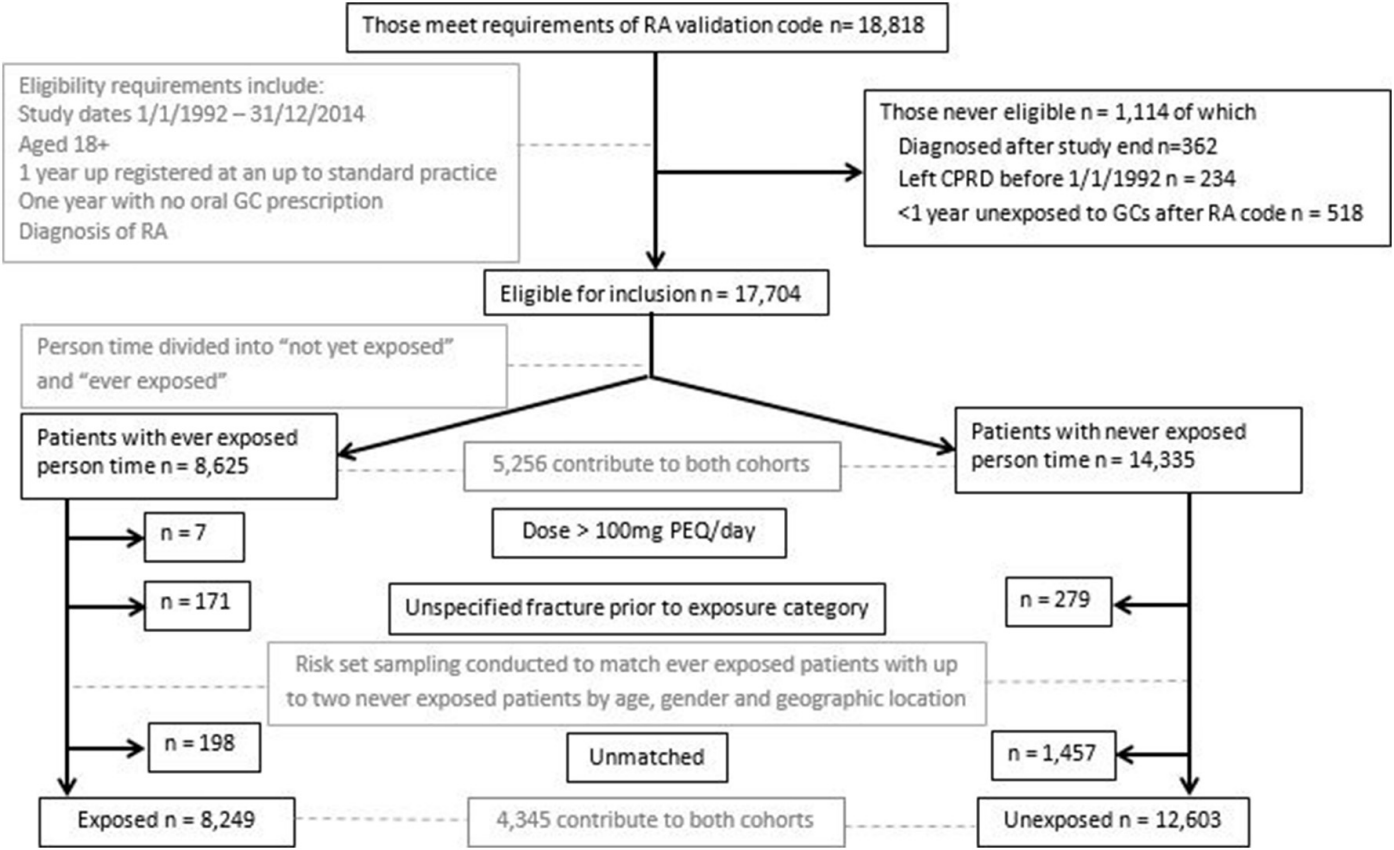
Flow chart of the selection of the study population.

Of 18,818 patients in CPRD with RA according to the validated algorithm, 16,507 were included in the final dataset. Fig. 1 describes the reasons for the exclusion of 12% of the patients.

Of 8249 patients exposed to oral GCs, all were matched to one as yet unexposed and 4289 (52%) were matched to a second as yet unexposed patient. For the first matched patient, 41% were the same age (with the remainder matched to someone within 5 years), 33% matched within practice and 90% matched within region. For the second matched patient, 30% were of the same age, 4% were in the same practice and 58% were within the same region. There were a total of 43,195 person years of ever exposed follow up and 63,452 person years of unexposed follow up time. Exposed patients spent a median of 4.2 years in the study whilst the matched unexposed patients spent only on average 3.7 years in the study. Table 1 shows the baseline characteristics of the exposed and unexposed patients with RA.

**Table 1:** Baseline characteristics of the exposed and unexposed cohorts.

Characteristic	Exposed	Unexposed^a^	*p*-Value
Number	8249	12,603	
Age in years, mean (SD)	60.8 (14.0)	57.7 (13.9)	**<0.001**
Gender (n, % women)	5624 (68.2)	8746 (69.4)	0.063
BMI at baseline			
<18.5	155 (1.9)	204 (1.6)	**<0.001**
18.5 to 25	1947 (23.6)	3045 (24.2)	
25–30	2140 (26.1)	3194 (25.3)	
30+	1808 (22.0)	2514 (20.0)	
Missing	2165 (26.4)	3646 (28.9)	
Baseline alcohol use			
Yes	6392 (77.5)	9805 (77.8)	0.746
Never	1141 (13.8)	1694 (13.4)	
Former	82 (1.0)	114 (0.9)	
Missing	634 (7.7)	990 (7.9)	
Follow-up in years, median (IQR)	4.2 (1.9, 7.5)	3.7 (1.4, 7.6)	**<0.001**
Number of GP contacts in previous 6 months, median (IQR)	9 (4, 14)	7 (4, 12)	**<0.001**
Charlson score			
1 (n, %)	5130 (62.5)	9292 (73.7)	**<0.001**
2 (n, %)	1823 (22.2)	2106 (16.7)	
3 (n, %)	722 (8.8)	719 (5.7)	
4 (n, %)	350 (4.3)	322 (2.6)	
≥5 (n, %)	190 (2.3)	164 (1.3)	
Drug exposure prior to baseline			
Immunosuppressives (n, %)	4719 (57.4)	5083 (40.3)	**<0.001**
Anti-osteoporotic therapies (n, %)	2618 (31.7)	2597 (20.6)	**<0.001**
Calcium + vitamin D supplements (n, %)	826 (10.1)	515 (4.1)	**<0.001**

Bold values indicates significance at P-value <0.05.

a Patients who start GCs will move to the exposed category upon their first exposure. The total number of patients in the unexposed group who switched into exposed is 4339.

Exposed patients were on average two years older than unexposed patients, more likely to be overweight or obese, had a higher Charlson comorbidity score and more likely to have a history of use of immunosuppressants, calcium and vitamin D or anti-osteoporotic therapies.

### 3.1 Patterns of oral GC use

Over 75% of exposed patients had at least one oral GC prescription of 10 mg PEQ/day or more, 14% had at least one prescription of 30 mg PEQ/day or more and 2.6% had a prescription greater or equal to 50 mg PEQ/day. Exposed patients were treated with oral GCs for on average 13% of the follow-up with a median dose of 9 mg (inter-quartile range 5, 21). Only 190 (2.3%) patients had continuous use from the first prescription until the end of follow-up, whilst 33% of the exposed patients spent at least one year on continuous oral GCs and 20% spending at least two years on continuous GC therapy.

### 3.2 Rates of fracture

There were 590 fractures in the exposed category and 516 in the unexposed category. This occurred over a total of 63,449 and 43,163 person years for the exposed and unexposed categories respectively. The rates of fracture were 136.7 and 81.3 per 10,000 person years for the same categories.

### 3.3 How models of risk attribution affect fracture risk

Table 2 shows the results of the analyses for the various models, including baseline rates of each group, unadjusted HRs and the final adjusted HR. The final model, with covariates with *p*-values < 0.2, is adjusted by baseline covariates of age, gender, alcohol category (missing included as a separate category), history of fracture, BMI category (missing included as a separate category), Charlson Co-morbidity Score, number of general practice contacts in the 6 months prior to baseline, injectable GC use, benzodiazepine use, opioid use and calcium/vitamin D use and the time varying covariate ever anti-osteoporotic therapy use. The covariates assessed for inclusion which did not have a *p*-value < 0.2 included baseline alcohol use, inhaled GC use, topical GC use, nasal GC use, rectal GC use, GC eye or ear drop use, immunosuppressant use, beta2 inhaler use, breast cancer treatments, prostate cancer treatment, anticonvulsant use and antipsychotic use. This was the same for all models.

**Table 2:** Comparison of common definitions of GC exposure using categorical models.

Model no.	Model⁎	Person years of follow up	Number of fractures	Fracture rate per 10,000 py	Unadjusted HR (95% CI)	Final model HR (95% CI)
1. Recency of use	Never exposed	63,448.7	516	81.33	Ref	Ref
	Current use	14,013.5	237	169.12	2.04 (1.75, 2.37)	1.43 (1.21, 1.68)
	Use in the past 1 day to 1 month	3669.3	63	171.69	2.13 (1.64, 2.76)	1.66 (1.27, 2.16)
	Use in the past 1–3 months	3189.7	36	112.86	1.41 (1.01, 1.98)	1.11 (0.79, 1.57)
	Use in the past 3–6 months	2544.8	28	110.03	1.34 (0.92, 1.96)	1.14 (0.78, 1.57)
	Use in the past 6 months to 1 year	3484.4	34	97.57	1.20 (0.85, 1.70)	1.02 (0.72, 1.46)
	Use > 1 year ago	16,260.2	192	118.08	1.36 (1.14, 1.61)	1.16 (0.97, 1.38)
2. Current dose^a^	0 mg and unexposed	63,448.7	516	81.33	Ref	Ref
	0.1 to 4.9 mg	4050.9	56	138.24	1.67 (1.27, 2.20)	1.12 (0.84, 1.49)
	5 to 9.9	6737.3	117	173.66	2.09 (1.71, 2.56)	1.44 (1.17, 1.77)
	10 to 14.9	1648.8	28	169.82	2.07 (1.42, 3.04)	1.52 (1.03, 2.23)
	15 to 19.9	476.5	14	293.78	3.63 (2.13, 6.17)	3.02 (1.77, 5.15)
	20 to 24.9	763.7	15	196.41	2.25 (1.34, 3.76)	1.87 (1.12, 3.15)
	25+	336.7	7	207.92	2.41 (1.14, 5.11)	2.10 (0.99, 4.44)
3. Cumulative dose	0 g	63,448.7	516	81.33	Ref	Ref
	0.1 to 0.9 g	16,936.0	199	117.50	1.43 (1.22, 1.69)	1.22 (1.03, 1.44)
	1 to 2.4 g	8745.3	101	115.49	1.41 (1.14, 1.74)	1.10 (0.88, 1.36)
	2.5 to 4.9 g	6901.6	107	155.04	1.87 (1.51, 2.30)	1.39 (1.12, 1.72)
	5 to 7.4 g	3608.0	59	163.53	1.96 (1.49, 2.57)	1.43 (1.08, 1.88)
	7.5 to 10 g	2297.6	49	213.26	2.53 (1.88, 3.41)	1.83 (1.35, 2.48)
	>10 g	4674.9	75	160.43	1.84 (1.43, 2.37)	1.32 (1.01, 1.71)
4. Peak dose	Peak dose 0 mg	63,448.7	516	81.33	Ref	Ref
	0.1 to 9.9 mg	10,466.4	144	137.58	1.67 (1.39, 2.01)	1.22 (1.01, 1.47)
	10 to 19.9 mg	8012.9	106	132.29	1.59 (1.29, 1.96)	1.17 (0.94, 1.45)
	20 to 39.9 mg	20,016.8	278	138.88	1.66 (1.43, 1.92)	1.37 (1.17, 1.59)
	40 to 59.9 mg	3879.5	53	136.61	1.60 (1.21, 2.13)	1.32 (0.98, 1.76)
	60 to 100 mg	787.85	9	114.23	1.36 (0.70, 2.63)	1.01 (0.52, 1.96)

⁎ The final model includes the covariates with a *p*-value < 0.2. Baseline covariates of age, gender, alcohol category (missing included as a separate category), history of fracture, BMI category (missing included as a separate category), Charlson Co-morbidity Score, number of general practice contacts in the 6 months prior to baseline, injectable GC use, benzodiazepine use, opioid use and calcium/vitamin D use and the time varying covariate ever anti-osteoporotic therapy use.

a In the current daily dose analysis, there were 353 fractures in 29,149.3 person years, a rate of 121.1 per 10,000 py when a patient categorised as exposed was not currently taking oral GCs.

**Fig. 2:**
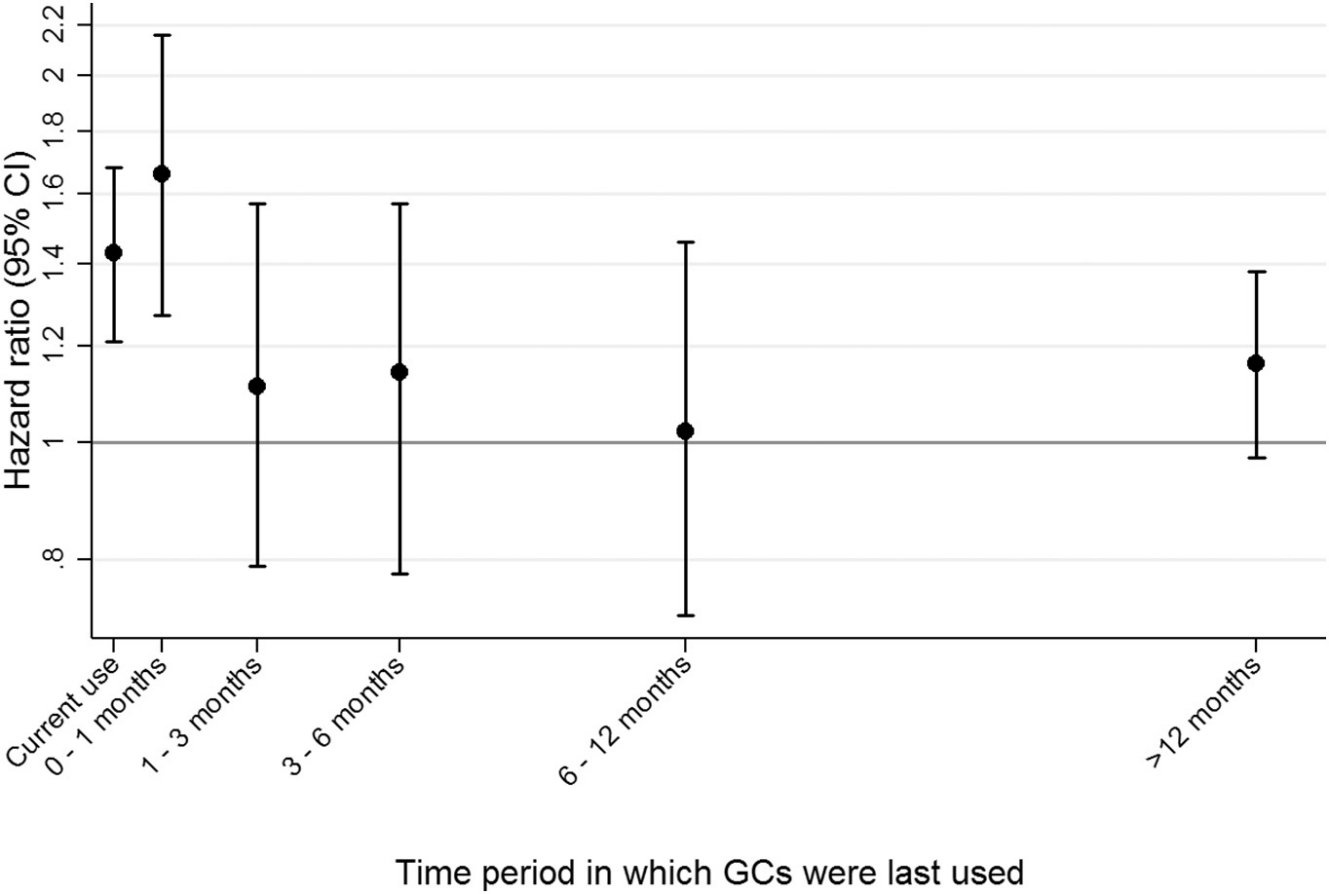
Risk of fracture in different time periods after discontinuation of oral GCs.

Model 1, which evaluated recency of exposure, found a significantly increased risk of fracture for patients currently using GCs with a HR and 95% CI of 1.43 (1.21, 1.68) compared to unexposed patients (Table 2 and Fig. 2). This risk then increased for patients who were exposed to GCs between 1 day and 1 month ago (HR 1.66 (1.27, 2.16)) before falling to 1.11 (0.79, 1.57) for use between 1 and 3 months ago. This risk remained non-significant for all further time windows.

Model 2 (Table 2) investigated the effect of current daily dose on the risk of fracture. Most doses above 5 mg PEQ/day had a statistically significant effect on the risk of fracture with HR starting at 1.44 (1.17, 1.77) for 5 to 9.9 mg PEQ/day before increasing to a peak of 3.02 (1.77, 5.15) for current doses between 15 and 19.9 mg PEQ/day. The HR then decreased for doses 20–24.9 mg PEQ (HR 1.87 (1.12, 3.15)) before increasing again for doses above 25 mg PEQ (HR 2.10 (0.99, 4.44)).

Model 3 (Table 2) investigated the effect of cumulative dose on the risk of fracture. As the dose increased, the risk of osteoporotic fracture generally increased although in an inconsistent manner. Small cumulative doses between 0.1 and 0.9 g PEQ increased the risk of fracture compared to non-use (HR 1.22 (1.03, 1.44)). Doses between 1 and 2.4 g PEQ had a smaller association (HR 1.10 (0.88, 1.36)) whilst higher cumulative doses between 7.5 and 10 g PEQ had a higher (HR 1.83 (1.01, 1.71)). The highest cumulative dose category of above 10 mg increased the risk of fracture to a lesser extent with a HR of 1.32 (1.01, 1.71).

The fourth and final model (Table 2) described the effect of the peak dose, i.e. the maximum dose a patient has had so far, on the risk of fracture. All peak doses below 20 mg PEQ increased the risk of fracture by about 20% (HR 1.22 (1.01, 1.47) and 1.17 (0.94, 1.45) for 0.1–9.9 mg and 10 to 19.99 mg PEQ). Peak doses between 20 and 59.9 mg PEQ increased the risk of fracture by about 35% (HR 1.37 (1.17, 1.59) for 20–39.9 mg and 1.32 (0.98, 1.76) 40–59.9 mg PEQ). Peak doses above 60 mg PEQ were not found to increase the risk of fracture (HR 1.01 (0.52, 1.96)).

With regard to the covariates, female gender increased the risk of fracture (HR 1.75 (1.50, 2.05). Each 10-year increase in age was associated with an increased risk of fracture of around 5%. BMI above 25 was associated with a lower risk of fracture: overweight patients (BMI 25.1–30) had a HR 0.79 (0.68, 0.93) compared to those with a BMI of 25 or below, whilst obese patients (BMI above 30) had a HR of 0.77 (0.64, 0.91). Anti-osteoporotic therapies, benzodiazepines and opioids increased the risk of fracture by 18%, 15% and 17% respectively, whilst use of injectable GCs, and calcium and vitamin D tablets did not show a statistically significant result. A full list of the covariates used in the final model with their HR and 95% CI for the current daily dose model are shown in supplementary table 2.

## 4 Discussion

This study has considered four different models for attributing fractures to patterns of oral GC exposure history including: recency of exposure, current daily dose, cumulative dose and peak dose. This is the first study to compare fracture risk estimates across these different oral GC exposure models, demonstrating that the attributes of dose, duration and recency of exposure each have an impact on fracture risk.

The recency of exposure model showed that exposure in the last month has a significant impact of fracture risk, with the risk declining and becoming statistically non-significant if GC exposure was stopped one month ago or further in the past. These results are in line with those previously produced by van Staa et al., [22] who found that the risk of fracture reversed towards baseline levels after discontinuation of oral GCs. This model has demonstrated that recency of exposure affects the risk of fracture, and that risks attributed to prior exposure can reduce with increasing time off treatment. This adds to previous work by demonstrating how risk of fracture changes in time windows not previously considered.

The current daily dose model showed an increase in fracture risk up to the 15–19.9 mg PEQ/day category and hence is once again in line with previous studies. [22,24] However it was also found that those on a dose above 20 mg PEQ/day had a lower risk than those on 15–19/9 mg PEG/day. A plausible explanation for this perhaps unexpected finding is that doses >20 mg tend to be used to treat flares in RA and are hence are only used for short periods of time. Lower doses may thus have a higher cumulative effect if used for longer durations. If we then consider prior literature on the effect of a cumulative dose there are inconsistent findings. Some studies showing an increased risk, [[26], [27], [28]] some showing an equivocal risk [29] and others showing a decreased risk of fracture. [30,31] In line with the results previously produce by de Vries et al., [28] the cumulative dose model in this paper showed an increased risk of with an increase in cumulative dose, although the magnitude of effect was lower in our study. The pattern of increasing risk with higher cumulative dose held true until the last category of >10 g PEQ where the risk of fracture reduced to 1.32 (1.01, 1.71) from a HR of 1.83 (1.35, 2.48) for cumulative doses between 7.5 and 10 g PEQ. Higher cumulative doses will be achieved with longer exposure histories, and thus it is possible that patients in the category >10 g PEQ exposure had some of their GC exposure a long time in the past. If recency is important, as our other models are suggesting, then the reduction in fracture risk for the highest cumulative exposure category may be explained by a lesser impact of doses taken a long time in the past. This reinforces the need to have a model that concurrently considers dose, curation and timing of exposure.

The attribution of peak GC dose to fracture risk has not previously been investigated. In this analysis, it was found that the risk of fracture was higher for those with peak doses between 40 and 59.9 mg PEQ and 20 and 39.9 mg PEQ compared to those with peak doses between 0.1 and 9.9 mg PEQ and 10 and 19.9 mg PEQ. This would be expected from the increase in risk found for current use at higher doses, assuming the exposure occurred sufficiently recently. The risk of fracture then reduced to no increase in risk for patients with a peak dose of 60 mg or more. This may be because of competing risks and a ‘perimortal bias’, as we have shown in other analyses [32], where high dose GC therapy is being prescribed for an indication such as malignancy at the end of life. Furthermore, the duration of use for such high doses tended to be short. A high peak dose may therefore not represent a significant cumulative dose, and it is also possible the peak dose may have been taken some time prior to the fracture.

Comparing the results to other published literature, fracture rates were lower than those reported by van Staa et al. [22] 200 per 10,000 person years and 130 per 10,000 person years for those exposed and unexposed, respectively, and lower than those reported by Brennan et al. [6] of 188 per 10,000 person years for women with RA. However, all fracture locations were considered in the analyses by van Staa et al. and Brennan et al., compared to six sites associated with known osteoporotic fractures in this analysis. This is supported by the results of Curtis et al. [7] where the risk of any fracture is 116.3 per 10,000 person years compared to 69.9 for fragility fractures which included the same locations as in this study. Should the result of Brennan et al. [6] be divided by the 1.66 relative rate found by Curtis et al., the rate of fragility fracture would be similar to those seen in this study.

With regards to the covariates, an increased risk of fracture was shown with anti-osteoporotic medication use. This may be considered unexpected since anti-osteoporotic medications are designed to reduce the risk of fracture. However, this likely represents confounding by indication [33,34] whereby patients given anti-osteoporotic medications start at an increased risk of fracture compared to those not treated. The beneficial effect of anti-osteoporotic medication can thus be lost within the increased risk prior to treatment.

Previous studies have shown heterogeneity in the results that could be explained by differences in study population, in the definitions of exposure and outcomes, of confounders, or the different choices of risk attribution model. Our study demonstrates, within a single cohort and with fixed exposure, outcome and confounder definitions, that the model by which fractures are attributed to GC exposure can have a major impact on the results. The range of models within our study highlighted the importance of higher dosage and more recent exposure (with the cumulative dose model considering in part duration of exposure) of oral GCs in increasing fracture risk. The models used in this study were not able to compare these aspects simultaneously, suggesting a new analysis combining these three aspects of dose, duration and recency of exposure is needed. One such method combining these three aspects is the Weighted Cumulative Exposure by Sylvestre and Abrahamowicz [35]. This method weights doses by how recently they were received using a data derived weight function enabling different patient specific patterns of use to be compared. This analytical approach has been used to examine the risk of serious infection [36] and incident diabetes [37] following GC therapy in patients with RA and could be applied to fractures which might validate the results of the current analysis.

Since fracture risk changes according to the chosen definition of oral GC exposure, the methods that are currently used in clinical practice to estimate the risk of fracture in oral GC users are limited by only considering one feature of GC exposure. These fracture risk calculators tend to consider oral GC use in a binary manner, for example, FRAX considers a GC user to be “currently exposed to oral GCs or have previously been exposed to oral GCs for more than 3 months at a dose of prednisolone of 5mg daily or more” [12]. As shown in this study, the strength of dose of oral GC exposure affects fracture risk. Kanis et al. [16] suggest a modification to FRAX to consider doses in the following categories: low (<2.5 mg), medium (2.5 to 7 mg) and high (≥7.5 mg). However, even this may not be sufficient. We have shown in Model 2 that does between 15 and 19.9 mg per day PEQ were associated with three-fold increase in fracture risk whilst a HR of 1.52 was seen for patients taking between 10 and 14.9 mg per day compared to non-use. Furthermore, whilst the FRAX tool considers a total duration of three months or more, the recency or timing of treatment is not considered. Our results suggest that risk declines significantly after one month of treatment. Until composite models can be developed and validated and embedded within such clinical decision support tools, the existing fracture assessment tools remain a useful way of assessing fracture risk.

There are limitations to this study that should be considered. First, there may be patients with a diagnosis of RA missed in this analysis due to exclusion criteria use in the algorithm for the definition of RA [20]. However, this should not affect the observed relationship between GC use and fracture for those included. Second, measures of RA disease activity are not available in primary care records. The number of GP contacts in the six months prior to baseline was used as a proxy for disease severity, although we accept this is an imperfect surrogate. Patients receiving GC therapy may well have higher disease activity which is itself associated with an increased risk of fracture, meaning there is the possibility of residual confounding. Third, vertebral fractures are often asymptomatic and hence are regularly missed in cohort studies. It has previously been estimated that approximately 30% of vertebral fractures are symptomatic [38] with the remainder going unnoticed unless the patient has imaging for a different reason. It is possible that patients taking GC therapy would be more likely to have back pain investigated given the known association with increased fracture risk, introducing a possible surveillance bias. However, asymptomatic fractures would not be more likely to be detected in those on or off GC treatment. Fourth, age matching was performed to within 5 years rather than the same year to enable more matches. This has led to a slight difference in age with those exposed to GCs slightly older than the unexposed. However, age was included in the model that should adjust for this difference. Finally, CPRD does not provide information about hospital prescriptions. This means there is likely to be misclassification of patients who have received oral GCs in hospital, but we would anticipate such prescriptions would be short-term given most long-term prescribing would be coordinated from primary care. There is also likely to be unmeasured confounding from anti-osteoporotic therapies, such as denosumab which are only prescribed in hospital, but we anticipate these would be prescribed in a small number of the current cohort.

## 5 Conclusion

In conclusion, this study shows that different aspects of oral GC exposure affect the risk of fracture. It is clear that dose, duration and recency of exposure all have an important impact of fracture risk, evidenced by the increased risk for more recent doses, higher doses and a longer duration spent on oral GCs. This level of complexity is not yet incorporated into fracture risk calculators. In the future, it may be possible to use and validate a more sophisticated modelling approach to generate a single risk estimate for any given pattern of GC exposure, and implement this algorithm within a computerised decision support tool. It is hoped that this analysis will encourage clinicians to consider the combined effects of dose, duration and recency of exposure when assessing the risk of fracture in patients being prescribed oral GCs.

## Conflicts of interest

DER and EMD declare no conflicts of interest. CC has received lecture fees and honoraria from Amgen, Danone, Eli Lilly, GSK, Medtronic, Merck, Nestlé, Novartis, Pfizer, Roche, Servier, Shire, Takeda and UCB outside of the submitted work. TvS has received lecture fees and honoraria from GSK and Pfizer outside of submitted work. WGD has received consultancy fees from Google and Bayer outside of the submitted work.

## Appendix A Supplementary data

Supplementary material Image 1
