# Supplementary material for: The limitations of using simple definitions of glucocorticoid exposure to predict fracture risk: A cohort study
Source: Bone. 2018 Dec;117:83–90. doi: 10.1016/j.bone.2018.09.004 (PMC6173307; doi:10.1016/j.bone.2018.09.004)
Supplement: Supplementary file 1 — Supplementary material [file mmc1.docx]

**Supplementary 1:**

Once all possible end dates of each prescription were had been estimated, using the data provided by CPRD about the quantity, prescribed number of tables per day and duration of prescription, the method to check for implausible results and fill in missing data was as follows.

1. Define implausible number of tablets prescribed per day and quantity to these were defined using the 99^th^ centile for each value and were defined as 10 and 210 respectively
2. Identify prescriptions with implausible quantity
3. Replace those with implausible quantity with
   1. mean of patient for that that specific drug, or if not available
   2. mean of all patients for that specific drug
4. Identify prescriptions with implausible number of tablets prescribed per day
5. Replace those with implausible number of tablets prescribed per day with
   1. mean of patient for that that specific drug, or if not available
   2. mean of all patients for that specific drug
6. Calculate the duration of the prescription in two ways where possible
   1. Physician defined number of days set in original prescription
   2. Quantity divided by number of tablets prescribed per day
7. Work out if these values are the same
   1. If so use the value, call it “real duration”
   2. Otherwise take mean
8. If “real duration” missing, take the patient’s mean duration of all other prescriptions for that specific drug
9. Calculate the stop date as the start day + “real duration”
10. Identify overlapping prescriptions of the same specific drug and calculate duration of overlap
11. If overlap of prescriptions is less than eight days, assume the prescriptions are continuous i.e. prescription lasts from the start of prescription 1 until the end of prescription 2. Keep number of tablets prescribed per day the same.
12. Identify any duplicate prescriptions of the same specific drug, start date and end date and remove
13. Calculate prednisolone equivalent dose per day of all prescriptions

**Supplementary 2: Covariates for the adjusted current dose analysis**

| **Covariate** | **HR (95% CI)** |
| --- | --- |
| Gender (Male reference group) | 1.75 (1.50, 2.05) |
| Age at baseline (per 10 years) | 1.05 (1.04, 1.05) |
| History of fracture | 1.16 (0.99, 1.36) |
| BMI <20  20 to 25  25.1 to 30  >30 | 1.31 (0.94, 1.83)  ref  0.79 (0.68, 0.93)  0.77 (0.64, 0.91) |
| Number of GP contacts in the past 6 months | 1.01 (1.00, 1.02) |
| Charlson score (per 1 unit increase) | 1.03 (0.94, 1.13) |
| Ever use of anti-osteoporotic therapies | 1.18 (1.04, 1.35) |
| Ever use at baseline of injectable GCs | 0.89 (0.76, 1.04) |
| Ever use at baseline of benzodiazepines | 1.15 (1.00, 1.31) |
| Ever use at baseline of opioids | 1.17 (1.02, 1.38) |
| Ever use at baseline of calcium and vitamin D tablets | 1.17 (0.95, 1.44) |
